# Epidemiology and Treatment Outcomes in Neonates with Esophageal Atresia: A 30-Year Population-Based Study

**DOI:** 10.3390/healthcare13040418

**Published:** 2025-02-14

**Authors:** Tanja Kovačević, Branka Polić, Joško Markić, Tatjana Ardalić Čatipović, Marija Bucat, Svjetlana Mikulić, Leona Žuvan, Zenon Pogorelić, Ranka Despot, Vanda Žitko, Julije Meštrović, Bernarda Lozić, Ana Jerončić

**Affiliations:** 1 Department of Pediatrics, University Hospital of Split, 21000 Split, Croatia; 2 School of Medicine, University of Split, 21000 Split, Croatia; 3 Pediatric Palliative Care Team, Health Center of Split-Dalmatia County, 21000 Split, Croatia; 4 Department of Neonatology, University Hospital of Split, 21000 Split, Croatia; 5 Department of Pediatrics, University Hospital of Mostar, 88000 Mostar, Bosnia and Herzegovina; 6 Department of Pediatric Surgery, University Hospital of Split, 21000 Split, Croatia; 7 Department of Research in Biomedicine and Health, University of Split School of Medicine, 21000 Split, Croatia

**Keywords:** esophageal atresia, prevalence, treatment outcome, postoperative complications, survival rate

## Abstract

**Background and objectives**: Outcomes of neonates diagnosed with esophageal atresia (EA), a rare congenital malformation, vary widely. Due to limited and fragmented data globally, major regional centers offer a crucial opportunity to better understand EA’s epidemiology and the management. This study aimed to address these gaps by determining total birth prevalence and early treatment outcomes of EA in southern Croatia. **Methods:** All EA cases (1991–2020) were retrospectively ascertained from medical documentation at the only tertiary referral center for EA in southern Croatia, with birth data collected from the entire background population. We collected data on neonates’ status and diagnosis, operative findings, early postoperative complications, and treatment outcome from this single center. **Results:** A total of 53 cases were identified, with an average total birth prevalence of 2.44 per 10,000 total births/year. No significant sex differences were found (*p* = 0.339), and most cases were complex Vogt 3B. The mortality rate dropped from 87 to 8% over a 30-year period (*p* < 0.001). The 1-year survival rate was 54% (95% CI 40–68%) for liveborns and 64% (50–79%) for liveborns who underwent surgery and intensive care. However, 16% of patients died before surgery due to hemodynamic instability, and among those operated on, high sepsis, pneumonia, and atelectasis rates were observed. **Conclusions:** EA prevalence in southern Croatia aligns with European data. Survival improved significantly after 2002, coinciding with a dedicated pediatric ICU and enhanced pediatric care training. Further advancements in early diagnosis and a multidisciplinary approach are needed to further reduce mortality. Strengthened postoperative infection control and optimized postoperative respiratory support are also crucial to minimizing complications.

## 1. Introduction

Esophageal atresia (EA) is a congenital malformation characterized by the complete absence of one part of the esophagus, often associated with a tracheoesophageal fistula (TEF) [1,2]. Based on the presence and location of a TEF, EA is classified into five types according to the Vogt and Gross classification [1,3]. The most common type is EA with distal TEF, accounting for 86% of cases, followed by pure EA (7%) [2,4]. EA is 2–3 times more common in twins and males [5,6,7] and frequently occurs in children with chromosomal abnormalities, such as trisomy 21 or 18 [2].

Associated anomalies occur in about 50% of EA cases [2]. These anomalies commonly affect various organ systems, including musculoskeletal, cardiovascular, gastrointestinal, and genitourinary systems (10–35%) [2]. EA is often linked to VACTERL association—a cluster of congenital anomalies including vertebral defects, anal atresia, cardiac defects, TEF, renal anomalies, and limb abnormalities [2,8]. Mortality in children with EA and VACTERL association remains high (12.5%) [9], influenced by the severity of the associated anomalies, prematurity, and pneumonia onset [10]. Tan Tanny et al. reported a threefold higher mortality rate in EA patients with VACTERL compared to those without [11].

Survival chances of children with EA have improved due to advances in neonatal intensive care and surgery [9]. Surgery is the mainstay of treatment. According to the Spitz classification—the most commonly used system for determining the survival after EA surgery [12]—survival is directly related to birth weight and the presence of major congenital heart defects (CHD). In the 1980s, Spitz et al. estimated a 97% survival rate for neonates with EA weighing over 1500 g without CHD (Group I), 59% for those with either major CHD or low birth weight (<1500 g) (Group II), and 22% for neonates with both major CHD and low birth weight (<1500 g) (Group III) [12].

In Europe, the total birth prevalence of EA is 2.43 (95% CI, 2.30 to 2.57) cases per 10,000 total births, ranging from 1.27 to 4.55 depending on geographical region [13,14]. This variation in prevalence may stem from several factors, including variations in study designs (population-based vs. single-center data), the scope of targeted populations (e.g., live births only, or live births and stillbirths, or live births, stillbirths, and miscarriage plus termination of pregnancy), diagnostic services, or genetic or environmental differences [7,15,16]. In addition, the current management of EA with TEF varies significantly in perioperative approaches across centers and is associated with notable postoperative morbidity and mortality [13].

In Croatia, epidemiological data on EA were initially collected in a single center—Zagreb—and later expanded to several regional hospitals through the European Database for Surveillance of Congenital Anomalies (EUROCAT) program [14]. Between 1986 and 2007, only 16 cases were reported, with a prevalence of 1.27 per 10,000 live births [13], whereas prevalence rates from 2013 to 2019 were highly variable (from 0 to 6.57 per 10,000 live births), highlighting gaps in the surveillance system [14]. Currently, no data on EA cases from southern Croatia were collected within the EUROCAT program or reported elsewhere. Croatia is a developed country with a healthcare system primarily funded through public health insurance, which ensures that public healthcare is widely accessible to its citizens. The majority of healthcare services, including pediatric surgery, are provided in public hospitals. Private healthcare options exist, but they are less commonly utilized for complex pediatric conditions due to the availability of specialized care within the public sector. While its healthcare system reflects Croatia’s status as a member of the European Union, it continues to face challenges such as resource allocation, staff shortages, and infrastructure modernization.

EA is a multifactorial disorder with no identified exclusive teratogens or inheritance patterns, requiring further investigation into the etiology and epidemiology of the disease [17]. In addition, effective care for this rare disease necessitates comprehensive regional and national epidemiological data, improved prenatal detection, and a better understanding of its clinical course. With better understanding about the condition, better resources can be allocated, like pediatric surgeons, nurses, and operating theaters from the government. For these reasons, a population-based study to evaluate the 30-year experience of neonates born with EA in southern Croatia was conducted. Our goal was to comprehensively delineate and accurately describe the types and prevalence of associated anomalies, as well as treatment outcomes.

## 2. Methods

### 2.1. Study Design

This retrospective observational study was conducted at the University Hospital of Split, Croatia, the tertiary referral center for births in southern Croatia and the only healthcare facility in the region equipped to manage and treat EA neonates. Data on births in the background population of southern Croatia were obtained from the Teaching Institute for Public Health, Split-Dalmatia County, which maintains these records. By utilizing a centralized and comprehensive data source, the study minimizes selection bias, ensuring that findings accurately represent the entire regional population rather than a selectively reported subset.

The study covers 30 years, from 1 January 1991 to 31 December 2020, divided into three equal periods. Given the rarity of the condition and the limited precision of short-term estimates, prevalence trends were analyzed in decade-based intervals, as performed in similar studies [13].

All cases that were diagnosed with EA at our institution during the observation period were included in the study, regardless of birth outcome (live births or stillbirths) or gestational age, provided the mother’s residence was in southern Croatia (Split-Dalmatia, Šibenik-Knin, Dubrovnik-Neretva counties). EA was diagnosed shortly after birth clinically and radiologically, with the type of EA being determined using the Vogt classification [1]. No exclusion criteria were applied.

Once the type of EA was determined, cases were further classified based on whether it was an isolated (only EA was present) or complex anomaly (EA with associated anomalies). Complex heart anomalies are defined as either cyanotic congenital heart defects requiring surgery or non-cyanotic defects requiring medical or surgical treatment. We additionally classified complex anomalies into two subcategories: one with and the other one without cardiac malformations.

The medical data were retrieved from available hospital records from the Department of Pediatric Surgery, the Department of Neonatology, the Department of Pediatrics, and the Department of Pathology of the University Hospital of Split. The data collected from hospital records for each participant with EA were date of birth, gestational age at birth, a plurality of births, sex, birth weight, Apgar score, type of atresia, operative finding, associated malformations, type of performed operation, early postoperative complications, and 1-year survival as treatment outcome.

### 2.2. Outcomes of Treatment

Given the long observation period, we chose to present treatment outcomes that are systematically and reliably recorded in the hospital’s records over the 30-year period. This includes long-term postoperative outcomes, such as 1-year mortality, and short-term outcomes, such as surgical complications. Additionally, for the period following the initiation of the pediatric intensive care unit (PICU), we presented data on the duration of intubation, length of postoperative stay in PICU, and length of hospital stay, as those were systematically collected after the establishment of the unit.

By focusing on these outcomes, we minimized inherent bias arising from incomplete or missing data, which may result from variations in medical record-keeping over time and affect the accuracy of findings.

### 2.3. Data Analysis

The distributions of qualitative variables using absolute and relative frequencies were described, whereas quantitative variables were summarized with the mean and standard deviation or the median and interquartile range, depending on the normality of the distribution. The normality of distribution was statistically tested with Shapiro–Wilk test.

The prevalence of EA was calculated per 10,000 births by dividing the total number of neonates diagnosed with EA (including both live births and stillbirths) by the total number of births. While the overall prevalence was presented for the observed period, to demonstrate changes in prevalence or mortality over time, we grouped the data into three 10-year periods (1991–2000, 2001–2010, and 2011–2020) and presented the rates for each period [13].

One-year mortality was determined as the proportion of liveborn neonates who died within one year of diagnosis, relative to the total number of liveborn neonates diagnosed with EA. Survival after the operation was calculated by dividing the number of operated neonates still alive after 1 year by the total number of children operated on for EA.

Trend analysis of prevalence across decades was conducted using the chi-square test for trend, while differences in sex distribution were assessed using the chi-square test. Additionally, 95% confidence intervals (CIs) for proportions were used to evaluate the significance of changes across decades. Data analysis was performed using MedCalc (version 23.0.6 MedCalc Software Ltd., Ostend, Belgium).

To ensure patient privacy, all data used in this study were fully anonymized prior to analysis. No personally identifiable information (e.g., names, dates of birth, or hospital identification numbers) was collected or included at any stage of the data analysis or reporting. This study was conducted in accordance with the principles of the Declaration of Helsinki and adhered to local regulations regarding the use of retrospective data.

## 3. Results

The study identified 53 neonates who were diagnosed with EA over the study period. Among 53 cases, 50 (94.3%) were live births, and 3 (5.7%) were stillborn. For seven children with a confirmed EA diagnosis, some of the medical data were missing from the archive.

### 3.1. Prevalence

The prevalence of EA in the three 10-year periods (1991–2000, 2001–2010, and 2011–2020) was 2.82, 2.37, and 2.03 per 10,000 births, respectively. The overall prevalence for the period of 1991–2020 is 2.44 per 10,000 (95% CI 1.78–3.10) births. While there appeared to be a decrease in prevalence with time, the trend was not statistically significant (*p* = 0.339).

### 3.2. Characteristics of Neonates with EA and Associated Pregnancies

Table 1 outlines the characteristics of neonates with EA and the pregnancies in which they occurred.

No significant difference was observed in the frequency between sexes, with a male-to-female ratio of 1:1.2 (*p* = 0.339). Regarding the type of atresia, most children were diagnosed with Vogt 3B—a blind esophageal pouch with a distal TEF. Vogt 2, characterized by a “gap” between two blind esophageal pouches without a fistula, was identified only sporadically. Additionally, most neonates presented with associated congenital anomalies, categorized as complex EA. Among these, 26 (76%) had two or more associated anomalies. Antenatal diagnoses of antenatal polyhydramnios were identified in only 11 cases. However, EA was not definitively diagnosed in any of these cases prior to birth.

Pregnancy complications were notable, with a high prevalence of preterm deliveries. Specifically, one-quarter of the children with EA were born prematurely, with a gestational age of less than 37 weeks. Among twin pregnancies, only two cases were recorded, and in both instances, only one twin was diagnosed with EA. Apgar scores were available for 34 neonates with EA, of whom 27% had an Apgar score (AS) ≤ 7, indicating moderate to severe distress at birth.

### 3.3. Surgical Treatment

**Figure 1:**
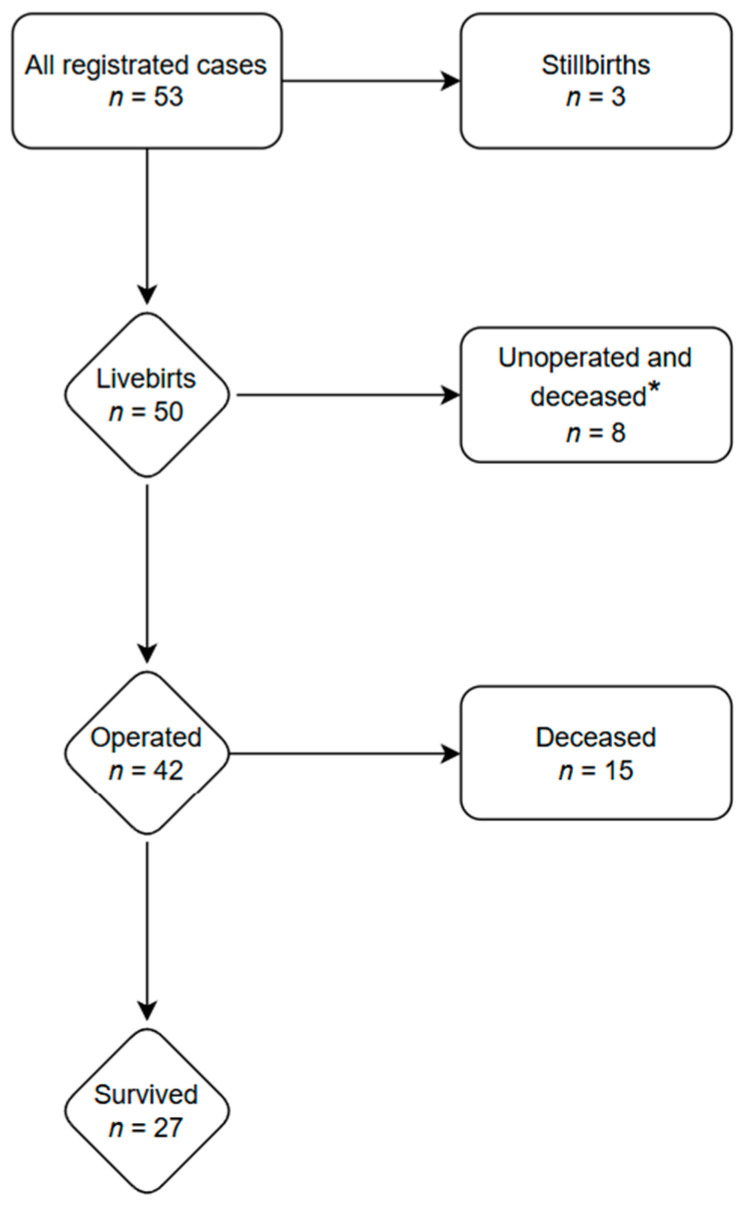
Patient flowchart. *—patients who had been hemodynamically unstable were not operated on.

Details of the surgical treatment are provided in Table 2, whereas the patient flowchart is shown in Figure 1. Among the live births, eight (16%) of the neonates were hemodynamically unstable and unable to undergo surgery, eventually leading to their early death, while 42 (84%) neonates underwent surgery.

For those who underwent surgery, the vast majority received primary esophageal reconstruction, with or without fistula ligation. Only two cases were initially managed with gastrostomy due to a long-gap atresia. Both neonates treated with gastrostomy did not survive the procedure, whereas those who underwent esophageal reconstruction had a 1-year survival rate of 68%.

The surgical management was performed on the first day of life in 36 cases (81%) and on the second day in 5 cases (16%). In one case it was not registered when the operation took place.

The most common early surgical complications were chylothorax or pneumothorax in five neonates or 12% of cases. Complications related to intensive care management, such as intrahospital sepsis and pneumonia, occurred more frequently than surgical complications. A total of 14 neonates had sepsis, and 8 developed pneumonia; 4 of them simultaneously had both complications. The incidence of sepsis was in as many as 33% of operated neonates, but no child diagnosed with sepsis died.

### 3.4. 1-Year Mortality and Survival of Operated Neonates

**Figure 2:**
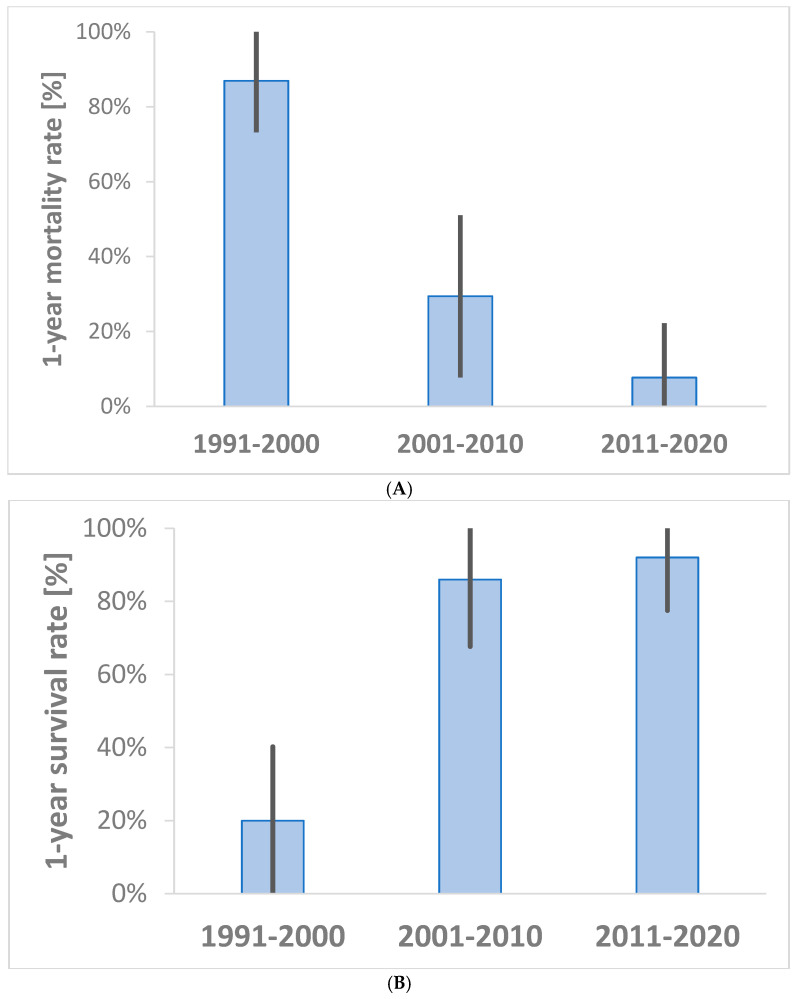
Trends in (**A**) 1-year mortality among neonates diagnosed with EA and (**B**) 1-year survival rate of operated neonates are shown with a 95% confidence interval across three consecutive ten-year periods.

The total 1-year mortality rate of liveborn neonates was 46% (95% CI 32–60%). There was a significant trend of declining 1-year mortality with time, with high values of 17 patients (85%) observed in 1991–2000 to just one patient (8%) in 2011–2020 (*p* < 0.001, Figure 2A). The overall survival rate of operated neonates was 64% (95% CI 50–79%) and increased throughout the three analyzed periods (*p* < 0.001, Figure 2B). When we analyzed whether the survival of operated neonates significantly increased after the establishment of the PICU at our institution, we found a marked improvement in survival rates from 2002, rising from three surviving neonates (20%) before the PICU to 24 (89%) following its initiation (*p* < 0.001). Documentation on postoperative outcomes, except for survival, was very limited during the pre-PICU phase, which hindered a more detailed comparison between postoperative outcomes between pre-PICU and PICU periods. In the PICU, the median duration of intubation was 8 days (range: 1–62), the postoperative stay in the PICU was 22 days (9–48), and the hospital stay was 37 days (25–275).

The 1-year survival rate decreased with increasing risk based on Spitz classification (Table 2, *p* < 0.001).

The relationship between mortality and congenital anomalies is detailed in Table 3, which presents the distribution of 1-year mortality rates by the systems affected by associated anomalies.

The highest mortality rate was observed among neonates with chromosomal abnormalities; three neonates were diagnosed with Edwards syndrome and two with Down syndrome. None of the affected neonates survived. Still, the burden of complex heart anomalies—defined as either cyanotic congenital heart defects requiring surgery or non-cyanotic defects requiring medical or surgical treatment—was even greater. All neonates with complex heart anomalies (9 cases, 36% of the total with associated anomalies) died, including both live births and stillbirths. In contrast, heart anomalies were the most frequently observed anomalies overall, appearing in 25 cases (74%). Importantly, all neonates with heart anomalies that were not classified as complex survived.

## 4. Discussion

This study offers a comprehensive perspective on the epidemiological, clinical, and surgical treatment aspects of EA, drawing on three decades of experience from the exclusive referral center for EA neonates in southern Croatia. Trends in mortality show substantial improvements over time, driven by enhanced treatment outcomes that reflect advancements in neonatal care, surgical techniques, and intensive care management. For the first time, the prevalence of EA was estimated for the population of southern Croatia, revealing an overall prevalence of 2.44 per 10,000 births. The findings underscore the stable occurrence of EA over this period.

The estimated prevalence in our region falls within the reported range for this rare disease in Europe, which varies from 1.27 to 4.55 EA cases per 10,000 total births in Europe [13]. The lowest prevalence in Europe, at 1.27 (95% CI 0.79–2.07) per 10,000 births, was reported over a 19-year period from 1987 to 2006 in Zagreb, northern Croatia. This was, however, significantly lower than the prevalence observed in our study for southern Croatia (*p* = 0.013). The difference in the prevalence of EA between northern and southern Croatia could be attributed to several factors, including unknown genetic and environmental factors, as well as methodologies employed in data collection and reporting, such as the organization of the healthcare reporting system. These factors likely interact and overlap, highlighting the need for further investigation to identify the precise causes of regional differences in prevalence. In our study, the prevalence of EA in the region did not change significantly during the 30-year period, suggesting that etiological factors remain constant over time.

According to the Vogt classification, which categorizes EA based on the anatomical relationship between the esophagus and the trachea, the most common type in our sample was type 3B, observed in 43 neonates (96%), followed by type 2, observed in two neonates (4%). This distribution aligns with data reported in the literature [1,13,18,19], which identifies type 3B as the most common type globally. Accurate identification of the EA type is essential for planning surgical interventions and anticipating potential complications.

A key clinical finding was the high prevalence of complex EA (64%), corroborating previous studies indicating that most neonates with EA present with associated anomalies [2,13,20,21]. Literature reports varying prevalence of associated anomalies in EA, from the percentage of 31.6% across Europe, 46.6% in France, 66% in Columbus, Ohio, to even 82% in Hawaii [13,20,21,22]. Differences between studies may be explained by methodological and population-based factors, but also potential, currently unknown etiological factors. The presence of complex forms of EA is often associated with an increased risk of long-term complications, including respiratory and gastrointestinal issues [23].

No statistically significant difference between the sexes was observed, although a higher number of neonates with EA were female. In other studies, this anomaly was more common in males [13,18,19]. The discrepancy in these results may be attributed to the relatively small number of EA cases as well as potential regional differences in the underlying populations.

The postnatal diagnosis of EA was mostly made on time—before the first meal, during the first day of life. Consequently, most of the surgical procedures were performed on the first day of life (36 cases, 81%). Diagnosing before the first feeding is standard practice in modern neonatal care, as it prevents aspiration, respiratory distress, and other severe complications that could arise if the anomaly goes underdiagnosed. The ability to make an early diagnosis is partly due to increased awareness among healthcare providers and adherence to standard diagnostic protocols. Timely diagnosis also facilitates quicker access to surgical intervention, ideally within the first 24 to 48 h of life. Early surgery can help to correct the defect before it leads to significant respiratory or nutritional challenges. Timely postnatal diagnosis is often a result of a coordinated approach between obstetricians, neonatologists, pediatric surgeons, and radiologists, highlighting the importance of a multidisciplinary approach in managing complex congenital anomalies like EA. However, antenatal diagnosis, specifically the identification of antenatal polyhydramnios, which was largely underreported in our records, highlights a potential area for improvement in regional care for these patients. Early and accurate antenatal diagnosis of EA could facilitate better prenatal management and preparation for surgical intervention and should be a focus of future research.

One of the most striking results of the current study is the dramatic decline in 1-year mortality from 87% in 1991–2000 to 30% in 2001–2010, and further to just 8% in 2011–2020. These results align with global trends in reduced neonatal mortality due to advances in prenatal diagnosis, optimized intensive care, and surgical techniques. Similar patterns have been observed in other studies [9,24]. Zimmer et al. analyzed the literature on the surgical outcome of EA for 80 years. The mortality rate after EA repair has significantly declined over time, from 100% in the presurgical era to 9% in recent years. Key reductions occurred during the 1940s-1990s, with mortality dropping from 81% to 16%, and further improvements were seen post-2000 due to advancements in surgical and neonatal care [24]. Also, Kassa and Lilja presented the notable decline in mortality rate over time in the VACTERL group: 20.0% (1973–1996) to 5.6% (1997–2018) [9]. The establishment of the PICU at our institution in 2002, combined with the structured education and training of medical personnel, is likely the primary contributor to the reduction in mortality rates in the treatment of children with congenital anomalies. Future pediatric intensive care staff were trained during 2000 and 2001 to work with critically ill neonatal patients, particularly for those after surgery at the Bambino Gesù Children’s Hospital in Rome, Italy, and subsequently at the University Medical Center in Ljubljana, Slovenia, and at the Children’s Hospital Zagreb in Croatia. Later, the staff continued their education in various European centers as well as at the Children’s Hospital of Philadelphia in the USA. This approach ensured the acquisition of critical knowledge and skills necessary for managing these vulnerable patient populations, ultimately improving clinical outcomes and setting a new standard of care. Before 2002, there was a lack of specialized neonatal intensive care facilities. Neonates with congenital anomalies were treated at a poorly equipped neonatal ward. The staff was not adequately trained; they lacked the necessary equipment and monitoring and did not have access to adequate facilities. This arrangement posed significant challenges, as the neonatal ward was not equipped to handle the unique needs of critically ill neonates.

According to Spitz’s classification, we divided operated neonates with EA into three groups in our study [12]. The lowest 1-year survival rate was observed in neonates classified as Spitz group III (birth weight < 1500 g with significant cardiac anomalies), whereas the highest, significantly better survival rate was observed in those > 1500 g without cardiac anomalies (Spitz group I). In comparison to other studies, we found a slightly lower 1-year survival rate in the first Spitz group, which normally has the best survival rate [2]. We interpret this as the fact that most of the children from that group who died were operated on before 2002, when a well-equipped and sophisticated PICU was not yet available at our institution. Similarly, this also applies to the mortality of neonates in Spitz’s group III, among other factors contributing to mortality in this group. The fact that there were no survivors with a birth weight below 1500 g and complex congenital heart defect emphasizes the extremely high risk posed by this combination of factors. Studies worldwide show exceptionally low survival rates in this group, although outcomes vary depending on the availability of specialized care and treatment protocols [2,12]. In the original study from 1994, the 1-year survival rate in group III was only 22% [12]. Recent studies report improved survival rates (approximately 30–40%) in centers with advanced neonatal surgery and intensive care. This improvement is primarily seen in neonates without severe congenital cardiac defects and/or chromosomal abnormalities like trisomy 18, where survival rates remain critically low, often below 10% [24,25,26].

Primary esophageal reconstruction with fistula ligation was the most common surgical approach (95%) and was associated with the best survival outcomes (68%). All children underwent surgery through a right lateral thoracotomy using an extrapleural approach. The fistula was identified, ligated, and divided, after which the esophageal pouches were anastomosed using single sutures over a nasogastric tube. During the observed study period, there were no changes in the surgical technique. However, neonates with long-gap atresia (gap ≥ 3 vertebral bodies) requiring gastrostomy experienced 100% mortality [25]. Long-gap EA is a complex congenital condition where the distance between the esophageal segments complicates surgical repair. The survival rate has significantly improved globally, with mortality now ranging between 5 and 20% depending on the presence of comorbidities and complications [25]. The high mortality rates for neonates with long-gap EA in our study, in the early 1990s, probably reflect the challenges of treating this complex condition at that time. Between 1991 and 2000, the overall mortality rate of neonates with EA of 87% further highlights systemic limitations in neonatal care and infrastructure at our hospital. The significant improvement to an 8% mortality rate between 2011 and 2020 reflects advancements in specialized neonatal intensive care and surgical techniques. This narrative demonstrates both the challenges of the past and the progress achieved over decades. Globally, advances in surgical techniques for the treatment of EA have significantly improved outcomes for affected children [27]. Traditionally, EA was corrected by a single-stage repair that involved anastomosis of the proximal and distal esophageal segments. However, new techniques, such as minimally invasive surgery, have reduced trauma and shortened recovery time. The introduction of video-assisted thoracoscopic surgery (VATS) has allowed surgeons to perform repairs with smaller incisions, reducing pain and complications [27]. In addition, advances in preoperative care, including nutritional support and respiratory management, have improved surgical success rates. Some centers are now using tissue-reinforced esophageal segments to repair EAs with long gaps, offering a promising alternative for cases where direct anastomosis is not possible. Improved imaging techniques, including 3D reconstruction, allow better visualization of the esophagus and help surgeons plan for complex repairs [27,28]. The use of advanced suturing materials and robotic assistance has further refined surgical precision. Overall, these innovations have dramatically improved the prognosis for children born with EA [27,28,29].

Our results underscore the critical prognostic role of associated anomalies, consistent with the findings from other studies [11,24,26,29]. Specifically, complex cardiac anomalies, chromosomal abnormalities (e.g., trisomy 18), and syndromic conditions like VACTERL were linked to the highest mortality rates. Neonates with isolated EA had far better outcomes, with a mortality rate of only 4%, consistent with previous research [11]. Neonates with non-complex cardiac anomalies in our study survived in all cases, suggesting that the nature and severity of the associated anomalies significantly influence prognosis.

The high incidence of postoperative sepsis and pneumonia is concerning, though these complications were not associated with mortality in our cohort. The findings emphasize the need for further improvements in postoperative care protocols to minimize these complications.

The significance of these data lies in the potential to identify and address regional disparities in the epidemiology and treatment of the disease. A deeper understanding of epidemiological data at both national and regional levels, along with treatment outcomes, could contribute to improved outcomes by providing valuable insights into the needs and the management of children with this anomaly. By analyzing treatment outcomes over a 30-year period, it provides insights into the strengths and limitations of current practices, highlighting areas for improvement. The findings can guide the development of evidence-based protocols, enhance multidisciplinary collaboration, and inform training for medical staff. Additionally, understanding trends in complications and outcomes can help anticipate challenges and optimize care for future patients, ultimately contributing to better long-term results.

This study has several limitations.

First, due to the retrospective nature of the data and a very long observation period, our choice of treatment outcomes was limited to what was systematically and reliably recorded in the hospital’s records over time. This resulted in a restricted set of maternal and neonatal variables and limited our ability to fully evaluate all the short-term neonatal outcomes of interest, potentially overlooking certain relevant factors. However, by focusing on selected hard outcomes, we minimized inherent bias associated with incomplete or missing data, which may arise from variations in medical record-keeping over time.

Further, although we only collected data from a single center, the center serves as the main referral center for births in the region and is the only healthcare facility equipped to manage and treat EA neonates. By utilizing a centralized and comprehensive data source on EA cases and on births in the background population, the study minimizes selection bias, providing robust estimates of the prevalence of EA over an extended period and making the study population-based. As for treatment outcomes, they are assessed in a single center and may be influenced by single-center bias.

We were unable to access data on spontaneous or elective pregnancy terminations, as these were not recorded in the available medical documentation. Consequently, the calculated prevalence of EA may be underestimated, as cases from these unregistered events were not included. However, since our prevalence estimates align with published literature, we do not anticipate that this will significantly impact our results.

Other potential limitations, given the rarity of EA, include missing or incomplete data from medical records. Nonetheless, research estimates, such as the stability of prevalence rates over decades, as well as the distribution of cases by Vogt category and mortality rates, which were consistent with findings from previous studies, suggest that these constraints did not have a significant impact on the results.

## 5. Conclusions

This study provides valid estimates on the prevalence, clinical characteristics, and treatment outcomes of EA in southern Croatia over a 30-year period. The prevalence of EA in the region is consistent with data from other European regions. As observed elsewhere, many neonates with EA presented with associated anomalies.

The mortality rates for EA decreased markedly over the three observed decades, reflecting substantial advancements in patient care and intensive care management. Notably, postsurgical complications were not linked to mortality, further highlighting the progress in treatment approaches. This provides a space for the ongoing need for continuous learning and knowledge renewal among medical professionals.

However, despite these improvements, high mortality rates persist in certain subgroups, such as high-risk Spitz groups and neonates with complex anomalies, underscoring the urgent need for innovative therapeutic strategies and enhanced complication prevention. The results of our study may help in designing treatment protocols and standardizing care with the aim of improving the treatment outcomes. The most pronounced unmet needs were identified in neonates with chromosomal abnormalities and complex cardiac anomalies, who exhibited the highest mortality rates. Addressing these challenges remains a priority for future research and clinical advancements.

## Figures and Tables

**Table 1:** Characteristics of neonates with esophageal atresia and associated pregnancies.

Characteristics	
**Pregnancies/delivery, *n* = 51**	
Delivery, *n* (%)	
Vaginal	28 (55)
Cesarean	23 (45)
Multiple pregnancies, *n* (%)	2 (4)
**Neonates, *n* = 53**	
Births, *n* (%)	
Live births	50 (94)
Stillbirths	3 (6)
Birth weight [g], mean ± SD	2720 ± 747
Gestational age, median (IQR)	39 (37–40)
Premature births (<37 weeks), *n* (%)	13 (25)
Apgar score ≤ 7, *n* (%) *	9 (27)
Sex, *n* (%)	
Male	24 (45)
Female	29 (55)
Vogt *n* (%) **	
Vogt 2	2 (4)
Vogt 3B	43 (96)
Associated congenital anomalies, *n* (%)	
Complex (at least one additional anomaly)	34 (64)
Isolated (no additional anomalies)	19 (36)

* Data were available for 34 neonates; shown is the valid percentage. ** Data were available for 45 neonates; valid percentages are shown. SD—standard deviation; IQR—interquartile range.

**Table 2:** Summary of surgical treatments and treatment outcomes.

Characteristics	*n* (%)
Surgical treatment status of neonates, *n* = 50	
Severely ill, not operated	8 (16)
Operation	42 (84)
Type of surgical treatment undertaken, *n* = 42	
Primary esophageal reconstruction ± fistula ligation	40 (95)
Initial gastrostomy	2 (5)
1-year survival by surgical treatment, *n* = 42	
Survived—primary reconstruction ± fistula ligation	27 (64)
Survived—initial gastrostomy	0 (0)
1-year survival by Spitz classification group, *n* = 42	
Group I (low risk) *, *n* = 30	24 (80)
Group II (intermediate risk) **, *n* = 10	3 (30)
Group III (high risk) #, *n* = 2	0 (0)
Complications	
Sepsis	14 (33)
Pneumonia	8 (19)
Atelectasis	5 (12)
Pneumothorax	3 (7)
Cardiorespiratory failure	3 (7)
Chylothorax	2 (5)
Pleural effusion	2 (5)
Anemia	2 (5)
Hyperbilirubinemia	2 (5)
Hepatorenal failure	1 (2)
Ileus	1 (2)
Anastomotic stricture	1 (2)
Pneumomediastinum	1 (2)
Fistula between the esophagus and thoracic cavity	1 (2)
Urinary tract infection	1 (2)
Necrotizing enterocolitis	1 (2)

* Group I—birth weight > 1500 g without a significant cardiac anomaly. ** Group II—birth weight < 1500 g or with a significant cardiac anomaly. # Group III—birth weight < 1500 g with a significant cardiac anomaly.

**Table 3:** The 1-year mortality among neonates and stillborn with EA, by associated congenital anomaly, *n* = 34.

Anomaly	*n*	Died (*n*)	1-Year Mortality Rate (%)
**Cardiovascular**	25	9	36
Complex cardiovascular *	9	9	100
Non-complex cardiovascular	16	0	0
**Chromosomopathies**	5	5	100
**Genitourinary**	6	4	67
**Skeletal**	4	2	50
**Digestive system**	4	2	50
**Head and neck deformities**	5	2	40
**Brain**	1	0	0
No associated anomaly	19	1	4

* Complex heart anomalies are defined as either cyanotic congenital heart defects requiring surgery or non-cyanotic defects requiring medical or surgical treatment.

## Data Availability

The data presented in this study are available on request from the corresponding authors.
